# Psychological resilience matters in the relationship between the decline in economic status and adults’ depression half a year after the outbreak of the COVID-19 pandemic

**DOI:** 10.3389/fpsyt.2023.1239437

**Published:** 2023-09-07

**Authors:** Jun Wan, Lin Liu, Yue Chen, Tianchen Zhang, Jun Huang

**Affiliations:** ^1^School of Economics and Resource Management, Beijing Normal University, Beijing, China; ^2^School of Social Development and Public Policy, Beijing Normal University, Beijing, China; ^3^College of Letters and Science, University of Wisconsin, Madison, WI, United States; ^4^School of Sociology, Central China Normal University, Wuhan, China

**Keywords:** psychological resilience, economic status, depression, COVID-19, propensity score matching (PSM)

## Abstract

**Background/objective:**

The outbreak of COVID-19 in China since 2019 has had a significant impact on the mental health of people in Hubei Province during the three-year pandemic period. Therefore, studying the prevalence of depression among the population of Hubei Province since the pandemic is of great significance.

**Methods:**

Based on opportunity and stress theory, we collected provincial-level data from Hubei (*N* = 3,285) to examine the impact of declining economic status on depressive symptoms and to investigate the moderating effect of psychological resilience during the period of economic adjustment.

**Results:**

We used propensity score matching to estimate the treatment effect of economic status decline on depression severity and confirmed the moderating effect of psychological resilience. We found that the more that an individual’s economic status declines, the more severe that his or her depressive symptoms become. Specifically, each unit decrease in economic status is associated with an increase of approximately 0.117 units in depression level. In addition, our results indicated that psychological resilience significantly moderated the relationship between economic decline and depression (−0.184*).

**Conclusions and implications:**

Our study confirms the role of economic status in depressive symptoms. Compared with traditional research on the relationship between economic status and mental illness, this paper expands the research regarding the two in the context of a major public health emergency. Furthermore, we suggest ways to improve people’s mental health following the pandemic.

## 1. Introduction

As the most extensive global pandemic encountered by human beings in the past 100 years, the COVID-19 pandemic has not only caused damage to people’ bodies but also greatly affected their mental health. China is one of the most severely affected countries in the world by the COVID-19 pandemic. In December 2019, a new coronavirus was reported in Wuhan, Hubei Province, China, and over the next 3 years, the pandemic spread rapidly across the globe, threatening the lives and health of people worldwide. The closer to the center of the crisis, the more people’s mental state is negatively affected by the social crisis (1). People are more prone to negative emotions such as loneliness, depression and anxiety. Depression is an affective disorder and is regarded as a mental disorder with depressed mood, hopelessness, and helplessness as the main symptoms (2). The factors affecting depression include individual factors, social factors, mental factors, and so on. Sudden major public health events have a significant negative impact on depression. The earlier study of the COVID-19 pandemic showed that the COVID-19 pandemic and the lockdown and quarantine policy resulted in a higher prevalence of depression and lower levels of mental health among the Chinese. A comparison with the results of previous cross-sectional studies can conclude that the level of mental health of Chinese people is deteriorating (3).

Today, although National Health Commission of the People’s Republic of China Announcement announced that the COVID-19 pandemic is almost over (4), the mental damage it caused remains, and people’s mental status has not improved significantly (5). One explanation for such a high prevalence of depression may be that the reduction in income and economy during the pandemic increased the social stress of individuals. According to the opportunity and stress hypothesis in social stress theory, people with lower economic status will experience more stress in their lives with limited opportunities to relieve stress. Thus they are more likely to suffer from mental disorders such as depression (6). During the pandemic, work stoppage, unemployment, and income disruption led to a decrease in the economic status of people in Hubei Province and increased social pressure, which led to increased rates of depression.

Given the wide range of mental health issues affected by COVID-19, it is important to identify protective factors for depression. Current studies have shown that protective factors for depression in the COVID-19 pandemic include social isolation, being female, living in rural areas, talking to parents, and hobbies (7). In addition to that, other studies have suggested that psychological resilience is also a protective factor for depression. According to the Richardson model, mental resilience can interact with external or internal risk factors (8), which means that it can protect individuals from negative events, especially disasters or health crises and reduce the risk of mental illness (8, 9).

Although the COVID-19 pandemic has had a broad impact on society, the mental status of the affected people still varies greatly, and the degree of depression varies greatly between different groups, which we are very concerned about. We sought to explore the relationship between post-pandemic changes in economic status and depression and to demonstrate the protective role of psychological resilience against depression during major public health emergencies.

## 2. Literature review

### 2.1. Depression under public health emergencies and its influencing factors

Sudden public health events will not only affect people’s physical health, but also have a certain negative impact on people’s psychology. Increased work stress, work stoppage, income disruption and financial strain have caused serious mental distress to the public, exacerbating symptoms such as depression (10). Numerous studies have shown that the COVID-19 pandemic is severely damaging people’s physical as well as mental health (10–12). The World Health Organization estimates that the pandemic has led to a 25%–27% increase in the prevalence of depression worldwide (13). Studies from Israel, the United States and other countries found that the general level of depression increased significantly after the pandemic, especially the depression level of the general population in the United States increased from 8.5% to 27.8% (14, 15), further demonstrated the impact of the COVID-19 pandemic on individual mental health. Among people affected by the pandemic, a study involving several different countries in Europe, Asia, and North America found that the incidence of depressive symptoms ranged from 14.6% to 48% (14). Another study also showed that the prevalence of depression in the Asia-Pacific region reached 34% (10). Of these, China, the first country to be hit by the pandemic, has a 29% prevalence of depression (16). Since the outbreak of COVID-19, all sectors of Chinese society have been severely affected, with an increase in the prevalence of depression among frontline healthcare workers, patients with new crowns, and the general public. A study of healthcare workers in Wuhan, China, found that 50% of participants experienced depression and more severe symptoms among first-line nurses fighting the virus (17). In addition, family members of frontline health workers were also affected: their depression prevalence was 12.2%, higher than that of the general Chinese population during the non-pandemic period (18). Moreover, the prevalence of depression among COVID-19 patients has increased due to the dual stress of mental pain and mental fear. One study found that 18.6% of mildly infected patients in China were depressed. Moreover, a survey of general residents who were quarantined at home due to the pandemic was also conducted and found that the prevalence of depression was significantly higher in the quarantined population in Shenzhen (6.21%) compared to the general population in Chongqing (3.7%) who were not quarantined (19). Hubei Province in China was the first region to be affected by the pandemic, and the people of Hubei suffered far more mental stress than other regions. A survey conducted during the COVID-19 pandemic showed that depression symptoms were most severe in Wuhan, Hubei Province, compared to the rest of the country, followed by the rest of the province (20).

The onset of depression may be influenced by a range of factors, including physical factors, personal characteristics, social factors, mental factors, and geographic factors. In terms of physical factors, sleep health is one of important conditions affecting depression, and the COVID-19 pandemic has been demonstrated to lead to deterioration in people’s sleep quality, as evidenced by difficulty falling asleep at night and increased daytime nap time (21). Previous studies have shown that depression occurring in the context of a major life event is associated with poorer sleep quality (e.g., difficulty falling asleep, daytime sleepiness, awakening from sleep, etc.) (22). Demographic characteristics are also one of the avenues that many scholars have used to explore the impact of the pandemic on depression. Several studies have shown that gender, age, marriage, income, and educational attainment also influence the prevalence of depression: women, young adults, unmarried, those with less than junior high school education, and those with lower economic status are more likely to be depressed (10, 20, 23, 24). In terms of social factors, sudden major events and social support can affect the prevalence of depression. In addition to the negative impact of the COVID-19 pandemic itself on depression, the use of social media is also an important influencing factor. There are two different views on the role of social media in the existing research, one of which is that the use of social media is a protective factor against depression for people in the pandemic and is an important source of social support. People obtain health information and emotional support from peers from social media, especially WeChat and thus effectively reduce the negative emotions brought by the pandemic (12). However, another survey from the United States suggests that people who did not have mental problems prior to the outbreak may have used social media in a counterproductive manner, with searching and viewing online searches or social media posts about the coronavirus, reports of significant changes in personal lives due to the outbreak, and perceptions that the virus posed a threat to the U.S. economy, personal health, or finances being significant factors in people’s distress (25). In terms of mental factors, long-term isolation and blockade policies can also have a negative effect on people’s mental situation, with public health restrictions leading to increased loneliness (26). Loneliness is an important contributor to depression (27). As the duration of the pandemic increases, people also gradually lose hope in ending the pandemic, and short-term concerns and fears are transformed into long-term mental emotions, and in these pandemics, concerns about personal and family health, as well as uncertainty about the future, may lead to depression (28). Geography also plays an important role in the increase in the prevalence of depression, with some studies suggesting that during public health emergencies, the psychological state of people in different regions may change from region to region, known as the “ripples effect.” That is, the closer an individual is to the center of the crisis, the higher the awareness of risk and negative emotions about the event (1). This has been verified by studies of the effects of the Wenchuan and Yushu earthquakes on the mental status of nearby residents. Hubei Province, the epicenter of the COVID-19 pandemic in China, was the obvious starting point for the “ripple effect.” However, it has also been suggested that there is no significant difference in depression prevalence between regions (10), which differs from previous studies.

### 2.2. Decline in economic status as an antecedent of depression

An investigation during the pandemic showed that income disruption and financial stress were risk factors for the prevalence of depression among countries in the Asia-Pacific region (10). The COVID-19 pandemic added uncertainty to the already unstable economic resources of low-income households (29). This makes their survival more difficult. The economic status of people in several countries have been affected globally: a study of the economic status of a group of people with underlying diseases in Bangladesh found that 46.2% of participants reported experiencing economic hardship during the COVID-19 pandemic and 12.3% lost their jobs (30); the study from Argentina also showed that more than half of the participants reported economic problems. Those who lost their jobs during the pandemic often faced financial problems (31). In China, 33.7% of households in Hebei Province experienced a significant decrease in income, while only 0.4% experienced a significant increase in income during the outbreak (32). There is an important difference between the two. Thus, decline in economic status occurs in several countries, and this has become one of the important factors contributing to the increase in individual depression levels after the pandemic. Previous studies have examined the relationship between social stress and mental illness. In 1989, Pearlin (33) proposed the social stress theory, which consists of three components: stressors, stress mediators, and stress responses. In subsequent studies, this model has been supplemented and developed by many researchers. Both Thoits and Aneshensel emphasize the importance of social status in terms of social and psychological stress (34, 35). In 2005, Christopher G. Hudson proposed the opportunity and stress hypothesis, which suggests that economic status is strongly negatively associated with mental illness, implying that lower economic status tends to increase individuals’ exposure to social stressors, thereby increasing their likelihood of developing mental problems (6). In conjunction with the research theme of this paper, we consider the pandemic and the negative effects of the pandemic as a source of social stress (36). We select the opportunity and stress hypothesis as the theoretical basis for exploring the association between economic status and mental illness. Sudden public health crises will, to a certain extent, affect people’s economic status. For example, once the economic status of a low-income family declines, the family members will inevitably be exposed to more social pressure, and they will be more prone to worry about their future lives, which will lead to an increase in the prevalence of depression. Therefore, we put forward the following hypothesis.

*H1:* The greater the decline in economic status is, the greater the negative impact is on depression.

### 2.3. The moderation of psychological resilience in depression

Psychological resilience has long been one of the key concepts in studying the psychological impact of public health crises on individuals. In most of the studies related to COVID-19, psychological resilience has been defined as a psychological trait with positive psychological qualities that enable individuals to effectively cope with stressful situations (8, 18, 37–39). Psychological resilience varies enormously across individuals, and people with different levels of psychological resilience tend to have different levels of resilience and ability to recover from stressful events. It has been shown that psychological resilience is a protective factor against anxiety, depression, and stress (40), moderates the negative effects of risk factors (41), and has a significant negative predictive effect on depression in particular (18, 42). This is corroborated by a study from China: psychological resilience moderated the negative effects of negative life events on depressive symptoms after an earthquake (20). People with lower psychological resilience have poorer mental health outcomes in disasters (18), whereas the higher the psychological resilience, the greater the person’s ability to resist depression and anxiety, and the less likely they are to experience elevated levels of depression in the face of an unexpected public crisis event. There have been numerous articles explaining the role of psychological resilience in the pandemic. Garmezy (41) proposed psychological resilience was mentioned in the study as a protective factor that has an important role in regulating the negative effects of risk factors. This is supported by a study from China in which psychological resilience moderated the negative effects of post-earthquake negative life events on depressive symptoms (20). Other studies have also shown that psychological resilience has a significant negative predictive effect on depression (18, 42). People with lower psychological resilience have poorer mental health outcomes in disasters (18). This may be due to the constant stress and sense of crisis caused by the outbreak. Examples include prolonged isolation, fear of infection, despair, fatigue, lack of resources, lack of information, economic loss, and shame (43). Richardson’s model further developed the protective model of psychological resilience, it assumed that protective factors (e.g., the character, trait, or situational premise of resiliency) and risk factors (e.g., contingencies, negative life events, and adversity, etc.) interact with each other in a balanced manner (44). During the COVID-19 pandemic, the risk factors increase significantly, and more protective factors are needed to balance them and maintain a state of mental equilibrium (8). The subject of this paper is the relationship between the decline in economic status and individual depression, so following the above theory, we consider the decline in economic status after the pandemic as a negative event and depression as a negative effect, and for psychological resilience, we will continue to follow the previous literature and use it to moderate the relationship between the two variables, exploring whether individuals with different psychological resilience will differ in the degree of depression. Therefore, we propose the following hypothesis:

*H2:* Psychological resilience significantly moderates the relationship between the decline of SES and depression.

In January 2020, Wuhan declared a “city closure” policy, which lasted until April of the same year. During this period, the spread of the unknown virus and the increase in the number of deaths brought great psychological pressure to the people of Wuhan, and it is crucial to study the psychological conditions of the people in Hubei Province. Currently, research on the psychological condition of the people in Hubei Province after the outbreak of COVID-19 pandemic mainly focuses on exploring the psychological state of the people during the pandemic. Less attention has been paid to depression after the end of the pandemic. In addition, due to differences in geographic location and economic structure, foreign studies may not be fully adapted to the domestic environment. Therefore, based on the above research background, this study will focus on the depressive state of people in Hubei Province after the pandemic, and use the opportunity and stress hypothesis and Richardson model to explore the effects of changes in economic status on depression under the regulation of psychological elasticity. It will provide a reference value for the future response to the mental health problems caused by sudden public health crisis events.

## 3. Methods

### 3.1. Sampling

During the period of the COVID-19 outbreak, from January 23 to April 8, Wuhan and other cities in Hubei Province were subjected to strict lockdown measures. This study draws on original research conducted by the School of Sociology, Central China Normal University, in June 2020, which coincided with a critical period of psychological adjustment for the residents of Hubei. Data were gathered through an online questionnaire administered during the lockdown period. This survey aimed to grasp the psychological and behavioral status of the population, their work and living conditions, and thus facilitate a thorough analysis of the various manifestations of post-epidemic syndrome and effectively promote the restoration of economic and social order. This survey encompassed various modules on mental health, family relationships and family life, social interactions and economic behavior, and online behavior and social mindset. In order to prevent participants from facing overly lengthy questionnaires, participants were asked to answer the basic module and were randomly assigned to one of the four above thematic modules. A total of 3,285 valid participants older than 16 responded to the thematic modules of depression. The sample comprised 54.43% males; the average age was 32.40 years old, and 34.58% lived in Wuhan City. The research received ethical approval from the ethics committee of the School of Sociology at Central China Normal University in China.

We utilized the trade union platform of Hubei to distribute electronic questionnaires to a target population of 14 million workers throughout the entire province. In order to minimize sampling bias, our initial focus was on workers aged 16 and above who resided in cities at the county level or higher within Hubei province. To ensure data quality, we implemented a filtering prompt in the first questionnaire item. We excluded responses with a duration of less than 5 min where the distribution of response times shows a noticeable truncation at the 5-min mark, and those that displayed logical inconsistencies. To reduce duplicate submissions, we employed measures such as IP address identification and account restrictions. Finally, to achieve a representative sample, we applied appropriate weighting techniques using population statistics provided by the Hubei Provincial Federation of Trade Unions.

### 3.2. Measurements

*Depression level:* We took depression as our dependent variable and adopted the World Health Organization Five-item Well-Being Index (WHO-5) to measure depression symptoms. The WHO-5 scale has adequate validity both as a screening tool for depression and as a measure of the severity of depression severity (45, 46). Participants were asked to rate their status from all the time (score 0) to never (score 5) over the previous 4 weeks, and the total score could range from 0 to 25. The content of the index is positive, with a total score of more than 12 defined as poor mental well-being. The WHO-5 has been found to have adequate validity and good construct validity in Chinese populations (47). In this survey, the overall Cronbach’s coefficient of the scale was 0.964.

*The decline in economic status:* According to Howell and Howell (48), we adopted decreased income to measure economic status decline as the independent variable. The participants were asked to what extent COVID-19 inflected their family income in the survey. We based the response and classified the participants into two mutually exclusive types: the economic status decrease group (coded as 1) and the control group (economic status increased or remained the same, coded as 0).

*Resilience:* Resilience as our moderator was measured using the Connor-Davidson Resilience scale (CD-RISC). The CD-RISC comprises three dimensions and 25 items, each rated on a 5-point scale (1–5), with higher scores reflecting greater resilience (49). The Chinese version of the CD-RISC showed good reliability and validity in the Chinese population (50). In this survey, Cronbach’s α was 0.986.

*Covariates:* Based on the literature review, we found potentially available explanatory factors for depression perception. At the individual level, we included several demographic factors (age, gender, education, party, household registration, job status, social status), physical factor (sleep health), psychological factors (interpersonal relationships, the strictness of lockdown), and social factors (critical negative events, exposure to pandemic information, and negative encounters during pandemic). Table 1 shows the meanings and measurements of the above factors.

**Table 1:** Covariate meanings and measurements.

Covariates	Meanings and measurements
*Demographic factors*	
Age	Age as of 2022.
Gender	Male or female.
Education	The number of years of education a person completed.
Party	Whether one was a Communist Party member.
Household registration	It was categorized into 4 levels (1 = countryside, 2 = town, 3 = rural–urban fringe, 4 = urban areas) depending on the distance to city center.
Job status	Job status in the previous three months: 1 = Had job, 0 = No job.
Social status	Self-report of perceived social status with 10 grades, from low (1) to high (10). Social status refers to a person’s position or rank within a social hierarchy or structure.
*Physical factors*	
Sleep health	The product of sleep time and sleep quality, and sleep quality was rated by participants from very bad (1) to very good (4).
*Psychological factors*	
Interpersonal relationship (with family)	The frequency of quarrels with children/spouse during the pandemic.
Interpersonal relationship (with pandemic prevention personnel)	Whether one has conflicts with pandemic prevention personnel.
Subjective feelings about strictness	Subjective feelings about lockdown policy.
Frequency of going out	Objective strictness of lockdown policy.
*Social factors*	
Critical negative events	Whether one has COVID-19 cases (close-contact cases, suspected cases, confirmed cases or death cases) in the family: 1 = Had cases, 0 = No cases.
Exposure to pandemic information	The average amount of time participants have spent searching for and reading pandemic information since the lockdown.
Encounters with Hubei citizens	The number of following things that participants have encountered: (a) seen comments on the internet or in chat groups that discriminate against or curse Hubei/Wuhan citizens; (b) refusal to be accepted by local government and communities when returning home during the Spring Festival; (c) being excluded when traveling, such as not being allowed to stay at hotels; (d) being ostracized and attacked by relatives and neighbors when returning home during the Spring Festival; (e) being rejected by one’s boss because of being Wuhan/Hubei citizens when returning to work; (f) being shunned and ostracized by colleagues because of being Wuhan/Hubei citizens after returning to work.
Fixed: city	An ordered categorical variable in terms of the distance to Wuhan, including Wuhan City, other cities in Hubei Province, Hubei/Anhui/Henan Provinces near Hubei, or other provinces in China.

### 3.3. Analytical strategy

We followed a two-step analytical strategy to empirically examine the association between the decrease group and the control group. In the first step, we performed a propensity score analysis to control for potential selection bias. We used a developed package—teffects psmatch—available in Stata software, version 17.0, to estimate the average treatment effect on the treated (ATET). The propensity score matching method utilizes terminology commonly used in experimental studies, such as treatment group and control group. The underlying logic of the propensity score matching method is rooted in the influential counterfactual framework developed by Rubin (51). In this framework, the propensity score represents the conditional probability of receiving treatment given the observed covariates. By estimating propensity scores and ensuring that the treated and control groups have similar scores, the observed covariates are effectively controlled for. Consequently, any differences between the treatment and control groups can be attributed to the receipt of treatment, rather than to the influence of observed covariates. This adjustment enables better control for confounding factors. We adopted a 1:1 matching strategy with replacement, estimated the *p*-score by a logit model, and set the default caliper (52). This is a relatively balanced parameter setting that neither overly restricts the sample size nor excessively loosens it, ensuring a balance between sample size and representativeness. Only the sample in common support was matched, which ensured that the propensity score values of the treatment group and control group have overlapping ranges. In addition, we analyzed the sensitivity of matching based on Imbens (53), examining the impact of confounding factors on the treatment variable and the outcome variable. We also conducted a heterogeneity analysis, investigating the extent of the impact within different subgroups based on our points. In the second step, we estimated an ordinary least-squares linear regression model and a multiple linear regression using income decrease as the key response. The goal is to understand the different effects of income decrease on the probability of depression levels among citizens after adjusting for a set of 17 covariables. Model 1 was our baseline model. Based on Model 1, Model 2 added demographic covariates, and Model 3 added all covariates. The matched columns show the compared results of estimates after applying sample weights depending on the number of matched times generated during matching. Finally, we checked the possibility of resilience as a moderator of the model.

## 4. Results

### 4.1. Descriptive statistics

Descriptive statistics are presented in Table 2 to summarize the characteristics of the sample and examine the distributions of variables. Overall, 60.49% of participants’ economic status decreased during the lockdown, whereas 39.51% increased or remained the same. The average resilience level was approximately 3.38. From the total column in Table 1, 32.24% of the sample had poor mental well-being; the average education years numbered 14.02; 27.21% were party members; 28.01% were Wuhan citizens in our sample, and 53.24% lived in the countryside far from the city center; and 7.82% did not have jobs the 3 months before our survey. Perceived social status was approximately middle (5.58 of 10). Only 5.05% of respondents did not have conflicts with pandemic protection personnel; almost half of them thought the lockdown policy was very strict, and 64.81% did not have a chance to go out. A total of 6.64% had COVID-19 cases in their family. On average, our respondents spent 2.46 h searching for or reading COVID information; each citizen encountered 1.34 negative incidents.

**Table 2:** Descriptive characteristics of participants, according to income decline, before and after propensity score matching.

			Unmatched					Matched				
	Total		1 = decrease		0 = control			1 = decrease		0 = control		
	Mean (%)	SD	Mean (%)	SD	Mean (%)	SD	*p* value	Mean (%)	SD	Mean (%)	SD	*p* value
Depression level (0–5)	1.76	1.44	1.87	1.39	1.61	1.50	−0.260***	1.76	1.42	1.60	1.45	−0.165**
≤ 2.4	67.76		65.58		71.11			66.23		71.65		
> 2.4	32.24		34.42		28.89			33.77		28.35		
Gender							−0.034*					0.004
Female	45.57		44.24		47.61			46.17		45.80		
Male	54.43		55.76		52.39			53.83		54.20		
Age (16–70)	32.40	9.63	31.09	9.11	34.42	10.1	3.332***	32.98	9.75	32.75	9.59	−0.230
Education years	14.02	2.84	13.87	2.79	14.25	2.91	0.376***	13.96	2.78	14.06	3.10	0.096
Party membership							0.091***					−0.007
Non-party member	72.79		76.40		67.26			70.05		70.73		
Party member	27.21		23.60		32.74			29.95		29.27		
Household registration							0.368***					0.000
Urban areas	17.05		20.38		11.94			13.19		15.35		
Rural–urban fringe	10.59		12.23		8.09			10.69		8.66		
Town	19.12		20.38		17.18			20.71		18.24		
Countryside	53.24		47.01		62.79			55.41		57.74		
Job status							0.031***					0.002
No job	7.82		9.06		5.93			7.39		7.22		
Had a job	92.18		90.94		94.07			92.61		92.78		
Social status (1–10)	5.58	2.14	5.44	2.13	5.79	2.13	0.359***	5.67	2.03	5.63	2.15	−0.042
Resilience	3.38	1.22	3.32	1.17	3.46	1.29	0.137***	3.39	1.21	3.38	1.29	−0.011
Quarreled with family							−0.099***					0.014
No at all	43.29		41.47		46.07			43.40		42.13		
Ordinary	44.60		44.34		44.99			45.65		46.85		
Very frequent	12.12		14.19		8.94			10.95		11.02		
Conflicts with personnel							0.015*					0.007
No conflicts	5.05		5.64		4.16			5.01		4.33		
Had conflict	94.95		94.36		95.84			94.99		95.67		
COVID-19 cases							−0.019**					−0.011
No cases	93.36		92.60		94.53			93.01		94.09		
Had cases	6.64		7.40		5.47			6.99		5.91		
Subjective feelings about strictness							0.047					0.030
Not at all	2.50		2.11		3.08			2.37		2.89		
Not too much	2.83		3.37		2.00			2.64		1.97		
Ordinary	7.46		8.10		6.47			8.84		6.69		
Relatively	32.94		33.42		32.20			31.27		32.55		
Very strict	54.28		52.99		56.24			54.88		55.91		
Frequency of going out							0.026					−0.034
Not at all	64.81		65.53		63.71			63.98		66.80		
Not too much	28.16		27.43		29.28			28.23		26.38		
Ordinary	5.72		5.99		5.32			6.33		5.12		
Relatively	0.94		0.75		1.23			1.06		1.18		
Very frequent	0.37		0.30		0.46			0.40		0.52		
Sleep health (0–40)	21.23	7.91	20.36	7.90	22.58	7.74	2.221***	21.06	7.91	21.60	7.86	0.534
Pandemic information (0–10)	2.46	1.66	2.50	1.66	2.38	1.66	−0.120**	2.38	1.61	2.44	1.70	0.060
Encounters (0–6)	1.34	1.45	1.43	1.43	1.22	1.46	−0.205***	1.28	1.37	1.35	1.57	0.068
City							−0.035					0.000
Wuhan	28.01		27.83		28.27			28.76		28.22		
Other cities in Hubei	40.52		39.51		42.06			38.79		41.08		
Henan/Hunan/Anhui	6.70		7.85		4.93			8.05		5.12		
Other provinces	24.78		24.81		24.73			24.41		25.59		
Total	3,285		1,987		1,298			758		762		

SD, standard deviation. *p < 0.1, **p < 0.05, ***p < 0.01. Wuhan is the capital city of Hubei Province. Henan/Hunan/Anhui are three provinces near Hubei.

Table 1 also compares the characteristics between the treatment group (decrease group) and the control group. The mean depression level in the treatment group was higher than that in the control group, both before and after matching. Before matching, the likelihood of being in the decreased group was greater for participants who lived in urban areas, quarreled frequently with family members, and had COVID-19 cases in their family compared with those in the control group. The likelihood of being in the decreased group was smaller for participants who were party members living in the countryside compared with those in the control group. On average, participants in the control group felt less depression, had healthier sleep, spent less time reading pandemic information, and encountered fewer negative events in life.

### 4.2. Multivariate results

Before estimating, we adopted the methodology of multiple imputations for missing values of the variable of social status, which were replaced by draws from the predictive distribution (54). We randomly generated several imputation values relying on the Bayesian model and data fit and used the mean imputation value as the unique value for matching and further analysis. Then, we checked the quality of propensity score matching according to the procedures. We conducted paired t-tests with the propensity-score-matched groups. The results showed that the difference between groups was not significant after matching, excluding the treatment variable (see the compared *p* values in Table 2). We also reported the variables’ normalized bias, according to which all of them in matched groups numbered less than 10%, and most t-tests did not reject the null hypothesis that there was no systematic difference between the treatment group and the control group (Table 3). In addition, only 25 observations were off support, meaning that we lost a few samples during matching.

**Table 3:** Balancing hypothesis testing showing the variables’ characteristics before and after matching.

Variables	Unmatched	Mean		Bias (%)	*t* value	*p* value
	Matched	Treated group	Control group			
Gender	U	0.558	0.524	6.8	1.9	0.058
	M	0.555	0.556	−0.2	−0.06	0.949
Age	U	31.086	34.418	−34.7	−9.83	0.000
	M	31.146	31.374	−2.4	−0.79	0.432
Education	U	13.870	14.246	−13.2	−3.71	0.000
	M	13.890	13.863	0.9	0.29	0.770
Party	U	0.236	0.327	−20.4	−5.78	0.000
	M	0.238	0.236	0.3	0.11	0.911
Household registration	U	2.940	3.308	−32.9	−9.11	0.000
	M	2.955	2.982	−2.5	−0.72	0.470
Job status	U	0.909	0.941	−11.9	−3.27	0.001
	M	0.913	0.913	−0.2	−0.06	0.955
Social status	U	5.436	5.795	−16.9	−4.72	0.000
	M	5.432	5.416	0.8	0.23	0.817
Resilience	U	3.323	3.460	−11.2	−3.16	0.002
	M	3.327	3.286	3.3	1.03	0.304
Quarreled with family	U	1.727	1.629	14.7	4.1	0
	M	1.722	1.758	−5.3	−1.6	0.109
Conflicts with personnel	U	0.944	0.958	−6.8	−1.89	0.059
	M	0.944	0.953	−4	−1.23	0.221
Covid cases	U	0.074	0.055	7.9	2.17	0.030
	M	0.073	0.058	6	1.86	0.062
Subjective feelings about strictness	U	4.318	4.365	−5.1	−1.44	0.150
	M	4.317	4.359	−4.6	−1.47	0.141
Frequency of going out	U	1.429	1.455	−3.8	−1.06	0.288
	M	1.429	1.420	1.3	0.42	0.675
Sleep health	U	20.356	22.578	−28.4	−7.94	0.000
	M	20.428	20.451	−0.3	−0.09	0.930
Pandemic information	U	2.504	2.384	7.2	2.02	0.044
	M	2.497	2.503	−0.4	−0.11	0.912
Encounters	U	1.426	1.221	14.2	3.98	0.000
	M	1.413	1.423	−0.7	−0.22	0.826
City	U	2.296	2.261	3.1	0.88	0.379
	M	2.299	2.282	1.5	0.48	0.633

U, Unmatched; M, Matched.

Table 4 provides the results of matching. The level of depression in the treatment group was 0.247 higher than that in the control group on average, indicating that decreased income could increase depression levels by 0.247 on average (*p* = 0.000, SD = 0.068). Sensitivity analysis showed that no variable was located near the contour. This finding indicates that there were no unobservable effects on the outcome variable and the treatment variable, decreasing the treatment by half. Thus, *H1* was supported. Table 4 also shows the heterogeneity results of matching. Compared to citizens staying in Hubei during the lockdown period, decreased economic status was more likely to result in depression of Hubei citizens staying outside of Hubei during the lockdown period ( $\beta_{\mathrm{non}-\mathrm{Hubei}}$ = 0.204* vs. $\beta_{\mathrm{Hubei}}$ = 0.163*). Compared with people in rural areas, people living in urban areas ( $\beta_{\mathrm{urban}}$ = 0.379*** vs. $\beta_{\mathrm{rural}}$ = 0.208**) experienced less depression due to economic status decline. Additionally, individuals who had COVID-19 cases (close-contact cases, suspected cases, confirmed cases or death cases) in their families were more likely to experience depression when there was a decline in economic status ( $\beta_{\mathrm{cases}}$ = 0.278* vs. $\beta_{\mathrm{no}-\mathrm{cases}}$ = 0.251***).

**Table 4:** Average treatment effect of income declines on depression level.

	Coefficient	AI robust std. err.	*z*	*p* > *z*	[95% conf. interval]	
Total	0.247	0.068	3.66	0.000***	0.115	0.380
In Hubei	0.163	0.086	1.890	0.058*	−0.006	0.332
Not in Hubei	0.204	0.123	1.660	0.097*	−0.037	0.445
Rural area	0.379	0.109	3.490	0.000***	0.166	0.592
Urban area	0.208	0.095	2.180	0.029**	0.021	0.395
Cases	0.278	0.144	1.930	0.053*	−0.004	0.559
No cases	0.251	0.070	3.570	0.000***	0.113	0.389

^*^p < 0.1; ^**^p < 0.05; ^***^p < 0.01.

We tested for homoskedasticity with the Breusch–Pagan/Cook–Weisberg test, which indicated OLS robust estimations in all cases except matched Model 1 (matched) to control for heteroskedasticity. We checked for potential multicollinearity issues by computing the variance inflation factor (VIF). The results for the mean VIF ranged between 1.00 and 1.20, and all individual VIFs are far less than 1.57, far less than values that would suggest any multicollinearity issue being relevant. To consider potential correlation across observations for districts within the same cooperative arrangement, we clustered our estimations by the unit of the city. The Durbin–Watson statistics of our models indicated no autocorrelation problems in unmatched models. After introducing propensity score weighting, matched models unavoidably exhibited a certain degree of autocorrelation. The Shapiro–Wilk test showed that some variables were not distributed normally. Therefore, we used the robust regression method to test the structural models.

Table 5 presents estimates of the average effect of income decrease on depression levels (standard errors in parentheses) with different specifications. The results seemed relatively robust, with coefficient estimates consistently positive and remaining significant after adding all covariates (Model 3, $\beta_{\mathrm{matched}}$ = 0.117**, *R*^2^ = 0.203). This outcome suggests that a greater decrease in income was correlated with a worse degree of depression symptoms. At the same time, the models suggest that people living near the city center, people who have stronger psychological resilience, people who encountered more negative experiences, people who felt lockdown policies were less strict, and people who had conflicts with pandemic protection personnel reported higher depression levels. Having COVID-19 cases in the family, having more time spent with pandemic information, having more frequent quarreling with family, and having worse sleep health were significantly correlated with higher depression after adjustment.

**Table 5:** Effect of income decline on depression level.

	Model 1		Model 2		Model 3	
	Unmatched	Matched	Unmatched	Matched	Unmatched	Matched
Income decline	0.262^***^	0.0564	0.251^***^	0.00915	0.171^***^	0.117^**^
	(0.0522)	(0.0509)	(0.0535)	(0.0533)	(0.0504)	(0.0487)
Gender			−0.00893	−0.118^**^	−0.00491	−0.113^**^
			(0.0509)	(0.0506)	(0.0475)	(0.0458)
Age			−0.000113	0.00921^***^	−0.00165	0.00220
			(0.00274)	(0.00281)	(0.00254)	(0.00251)
Education			0.0149	−0.0101	0.00690	−0.0216^**^
			(0.0101)	(0.0100)	(0.00917)	(0.00906)
Party			0.0325	0.119^*^	−0.0425	0.00459
			(0.0614)	(0.0624)	(0.0572)	(0.0565)
Job status			0.0343	0.143	−0.0958	0.0121
			(0.111)	(0.112)	(0.104)	(0.103)
Household registration			−0.0512^**^	−0.0678^***^	−0.0551^**^	−0.0727^***^
			(0.0248)	(0.0245)	(0.0233)	(0.0225)
Social status			−0.00858	0.0596^***^	−0.0180	0.0407^***^
			(0.0150)	(0.0140)	(0.0137)	(0.0127)
Resilience					0.386^***^	0.425^***^
					(0.0211)	(0.0192)
Quarrel with family					0.129^***^	0.183^***^
					(0.0360)	(0.0361)
Conflict with personnel					−0.295^***^	−0.285^***^
					(0.0937)	(0.102)
COVID-19 cases					0.207^**^	0.246^***^
					(0.0839)	(0.0720)
Subjective feelings about strictness					−0.135^***^	−0.148^***^
					(0.0266)	(0.0262)
Frequency of going out					0.0319	0.0682^**^
					(0.0345)	(0.0340)
Pandemic information					0.0323^**^	0.0263^*^
					(0.0150)	(0.0158)
Sleep health					−0.0284^***^	−0.0324^***^
					(0.00317)	(0.00320)
Encounters					0.0665^***^	0.0803^***^
					(0.0171)	(0.0163)
Other cities in Hubei	−0.0102	−0.190^***^	−0.0319	−0.181^***^	−0.0585	−0.162^***^
	(0.0616)	(0.0635)	(0.0629)	(0.0650)	(0.0591)	(0.0595)
Henan/Hunan/Anhui	−0.0925	0.0648	−0.126	0.0463	−0.104	0.00217
	(0.110)	(0.109)	(0.114)	(0.116)	(0.0999)	(0.0986)
Other provinces	−0.108	−0.282^***^	−0.141^*^	−0.291^***^	−0.0415	−0.209^***^
	(0.0712)	(0.0710)	(0.0757)	(0.0762)	(0.0705)	(0.0686)
_cons	1.641^***^	1.797^***^	1.633^***^	1.441^***^	1.748^***^	1.637^***^
	(0.0590)	(0.0571)	(0.235)	(0.224)	(0.267)	(0.251)
VIF	1.00	1.00	1.19	1.20	1.16	1.19
B-P/C-W Test *p* =	0.019**	0.1925	0.000***	0.004***	0.000***	0.000***
Durbin–Watson	1.991	0.943	1.990	0.943	1.978	0.9578
*N*	3,285	3,260	3,285	3,260	3,285	3,260
*R* ^2^	0.009	0.008	0.011	0.021	0.148	0.203

Standard errors in parentheses. ^*^p < 0.1, ^**^p < 0.05, ^***^p < 0.01.

### 4.3. Moderate effect

**Figure 1:**
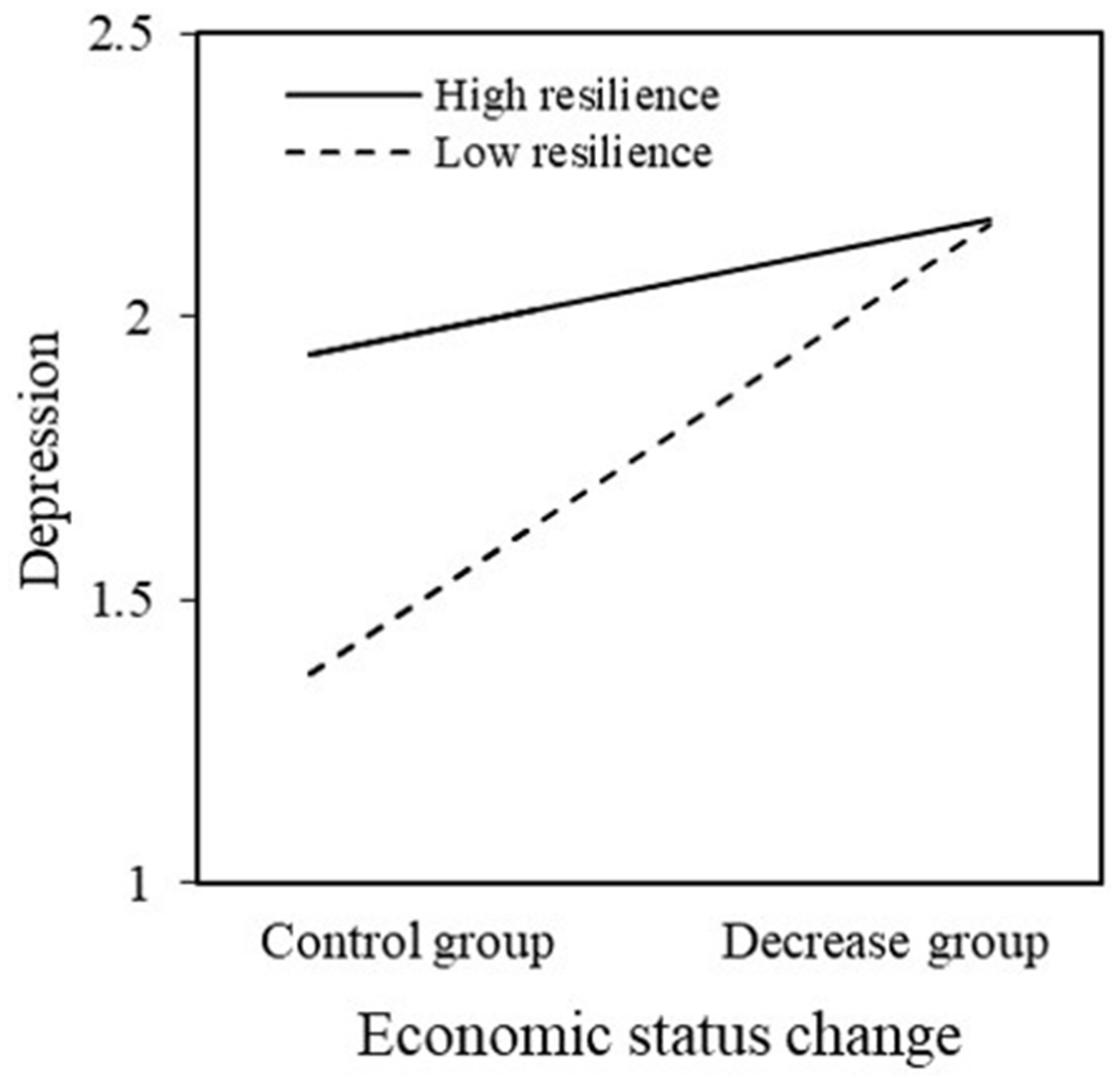
Moderate effect.

Next, we tested the moderating effects by adding the interaction of income decline and resilience in Stata software, version 17.0. Our goal was to further investigate the boundary condition of when the income decrease of the public influenced its depression level. We found that the estimated effect of income decrease on depression was significant (*β* = 0.264***, SD = 0.063, *p* = 0.000), and the interaction was significant (*β* = −0.184*, SD = 0.100, *p* = 0.066). This finding suggests that resilience as a moderator could weaken the relationship between decreased income and depression levels, indicating that the depression level caused individuals’ economic decline will diminish as psychological resilience increases. Thus, *H2* was supported (see Table 6 and Figure 1).

**Table 6:** Estimation of moderate effect.

	Coefficient	Robust std. err.	*t*	*p* > *t*	[95%conf. interval]	
Income decline	0.264	0.063	4.20	0.000	0.141	0.388
Resilience	0.563	0.079	7.09	0.001	0.407	0.719
Interaction	−0.184	0.100	−1.84	0.066	−0.379	0.012

## 5. Discussion

Our study revealed that, for participants who reported a decrease in economic status, an increase in depression levels was discovered compared to those with a constant economic status. Each decrease in the unit of economic status raised the level of depression by approximately 0.117 units. In addition, the results of this study demonstrate that psychological resilience significantly moderates the relationship between a decline in economic status and depression (−0.184*). In short, both of the hypotheses that we proposed were strongly supported.

### 5.1. The vital role of economic status in mental health indications

We first hypothesized that when experiencing a more severe economic status decline, individuals’ depression levels will be worse impacted. Consistent with previous studies (55, 56), our findings suggested that a more severe economic status decrease caused greater depression in individuals after a huge global crisis, such as the COVID-19 pandemic. A large proportion of the general population suffered from the suspension of work activities or was forced to work remotely during the pandemic outbreak. Some individuals even experienced the loss of job positions due to the economic recession (55). Under chronic job insecurity and financial threat, individuals have less sense of control and may experience more concerns about maintaining current living standards, which are significant risk factors for developing depression. Our findings further justified that economic standing could be an influencing factor in mental health status. Although existing studies have made significant contributions to understanding SES changes and depression under public health emergencies, we consider it essential to investigate whether a decrease in economic status would lead to depression in people living in the region where the pandemic originated and was most influenced at the beginning of COVID-19 specifically. The Hubei Province of China, with the center of Wuhan City, was where the coronavirus was first discovered and was affected the most due to a lack of knowledge about the virus transmission route and protection methods from infection.

### 5.2. The moderating effect of psychological resilience

Our second hypothesis of the moderating effect of psychological resilience was also supported by the findings. The psychological resilience level of participants in our sample was 3.38 on average. Our findings demonstrated that the negative influence of economic status decline on depression is lower in individuals with a higher degree of resilience. This result is in line with prior studies regarding the protective effects of resilience on mental well-being (39, 57). A study conducted in Wuhan, China (39), during the first outbreak of the pandemic also concluded that resilience is negatively associated with anxiety and depression in patients with less severe infection. On the one hand, with a higher level of resilience, individuals living in the pandemic control region have higher psychological resources to confront the negative mental influences brought by the decline of economic status. On the other hand, the decline in economic status could negatively influence the level of psychological resilience since income has been proven to be a significant indicator of the resilience of individuals (40). Therefore, when suffering a decline in economic standing, individuals may have less resilience as personal resources to protect them from developing depressive symptoms.

### 5.3. Other findings

According to the ripple effect model, individuals have higher risk perceptions and negative affection for a crisis when they are closer to the center of it (1). Nevertheless, apart from what most of the crisis literature and the ripple effect suggested, our findings demonstrated that, for residents who were not in Hubei during the pandemic outbreak in January 2020, their depression level was influenced even more by a decrease in economic status compared to that of residents who lived in Hubei at the time. A possible explanation could be that working and living in a city other than their home, individuals might face more financial stress regarding affording household expenses, paying housing rents, etc. When feeling that their economic status is greatly influenced by a global health crisis that is uncontrollable by individual forces, migrant workers are more likely to suffer from negative psychological well-being since they encounter more stress in life and have less support from family and friends. Therefore, our findings point out the need for attention and support, not only to residents who lived in the center of a crisis but also to the migrant population originating from the crisis center. The numerous cases of financial loss and negative mental health outcomes of Hubei residents and migrant Hubei residents during the pandemic should receive more attention from the government and the public since these people encountered relatively more detrimental influences compared to those in other regions. Although Hubei people who work and live in places other than their hometowns were previously ignored by researchers, our study suggests the latent risk of depression in this population. More investments in attention and resource allocations to this group in future crises that could lead to large-scale economic regression and psychological distress in people are necessary.

Moreover, our results demonstrated that the effect of decreased economic status on depression symptoms was also determined by the place of residence. For participants living in rural areas, an objective decrease in their economic standing led to a worse level of depression compared to those who live in urban areas. This outcome could be attributed to worry about a lack of effective medical resources or treatment in rural areas for potential infection. Additionally, whether one’s self, one’s family or one’s relatives were in close contact with infected cases, infected, or died due to infection is another variable that differentiated the influence of economic status decline on the development of depressive symptoms. Participants who reported having COVID-19 cases in their families showed more deteriorated depressive levels when their economic status decreased during the pandemic. This finding could be explained by discrimination against infected patients during the outbreak. Due to the lack of knowledge and effective treatment methods at the beginning of the pandemic, being infected could lead to consequences for long-term treatment under quarantine. Although patients recovered, they could face unemployment because of workplace discrimination. Therefore, being infected or having a family member who tested positive for COVID-19 could result in a decrease in household income and negatively influence one’s economic status.

### 5.4. Limitations and future studies

Although the present study provides some meaningful initial evidence to understand the impact of economic status decline on the mental well-being of the public during the postpandemic era, several limitations should be stated clearly to clarify the effective implications of our findings and the development of future research. First, our data were collected through an online questionnaire based on a trade union platform, which lacks representativeness compared to random sampling. However, we took various measures to reduce sampling bias. Second, our sample was between 20 and 40 years old and was recruited from the region where the first outbreak of COVID-19 occurred, which is the Hubei Province of China, instead of nationwide or globally. Therefore, the results of this study are not generalizable to a broader population with different ages, cultures, or severities of pandemic impacts. Third, we adopted WHO-5 as our measurement tool for depression, even though it is commonly regarded as a screening tool for detecting depression rather than reflecting the severity of depression. However, due to its cost-effectiveness, non-invasiveness, and limited evidence supporting its use for assessing depression severity (45), we decided to include WHO-5 in the questionnaire as a means of assessing depression. Nevertheless, although these inevitable factors limited the present study, our study still provided novel and meaningful contributions in that our data comes from a large population in the region the COVID-19 was first discovered.

Future research studying the influence of economic status changes on depression could focus on more diverse and broader populations. For example, researchers could investigate how a decline in household economic status affects the depression levels of children or elderly people from Western cultural backgrounds in different stages of infectious disease outbreaks.

### 5.5. Implications

It is worth mentioning that, unlike existing studies regarding the immediate influences of SES decreases on psychological well-being, the current study focused on investigating the relationship between SES and depression approximately half a year after the world’s first outbreak of the COVID-19 pandemic. Studying the postpandemic period is essential since valuable results generated from these studies could guide us in formulating a more effective and precise plan to confront the detrimental consequences of crises and to construct a more extensive and systematic risk response plan to lower the threat when future crises occur. The Guiding Principles for Emergency Psychological Crisis Intervention in the Outbreak of Novel Coronavirus Pneumonia, issued by the National Health Commission during the pandemic, note that the public in the pandemic area is the fourth-level target population for psychological interventions, suggesting that communities should pay attention to the mental health status of residents and meet the needs of residents (4). Nevertheless, reconstruction practices for communities in Hubei and interventions for Hubei residents should also be greatly promoted in the postpandemic era to decrease the negative influences caused by the global health crisis. Effective psychological interventions and services should be provided to the public to prevent detrimental experiences of stress, trauma, and emotional distress from developing into chronic psychological disorders such as PTSD, anxiety, and depression.

Additionally, the results of the present study point out the necessity to improve the level of resilience of the public regarding preventing psychological detriments from coming with future crises. Higher resilience possibly helps to prevent negative affect from developing into chronic mental disorders. Therefore, governments should invest in exploring effective resilience improvement programs to help individuals to develop a higher level of resilience, which could be beneficial for them to withstand various threats, not only at the macro scale but also at the individual level. Unlike survivors of other crises, individuals during the COVID-19 outbreak were required to be socially isolated from others, which led to the loss of social support and networks to a great extent (58). Social support, as an aspect of interpersonal resources, has proven to be an essential factor in increasing resilience (59, 60). Due to the loss of social connections during the global health crisis, individuals could have less support helping them to remain positive to confront threats that come with crises. Hence, social support is inevitably a vital component to consider when relevant governments and social sectors formulate crisis response strategies and policies. Cutting the social connections of individuals should be prevented to a great extent to maintain individuals’ sources of obtaining social support and cultivating resilience. In addition, policy-makers and relative social sectors should expand methods to provide more social support to individuals during crises. For example, crisis interventions and psychological counseling services could be effective ways to socially support individuals during extreme traumatic events.

Our study also provides empirical evidence to support theories. First, consistent with the Opportunity and Stress Hypothesis (6), our findings suggested that a greater decrease in economic standing generates worse depression levels in individuals. The Opportunity and Stress Hypothesis claims that people with more disadvantaged SES have less social capital to confront crises in life. When individuals experience financial loss during the pandemic, they could fall into a situation in which fewer resources can be utilized to face the negative consequences of the pandemic. Economic challenges can also become a chronic stress situation in families, which can generate distressing thoughts about paying household expenses and lead to fear, anxiety, and uncertainty regarding one’s ability to maintain the current standard of living (55). Being restricted and isolated at home for two and a half months, residents of Hubei could feel insecure about their jobs and financial situation, increasing their risk of depression during the infectious disease outbreak. Second, the current study also provided support for the protective model of psychological resilience, which proposes the protective effect of stress resistance against the development of psychopathology under stress generated from risk factors (41). Stress resistance, as a vital assessing element of resilience and coping, represents individuals’ competence to confront the negative effects of stressful events. Possessing a higher level of competence when facing stressors in life, individuals have higher resilience to protect themselves from developing negative psychological outcomes. In line with this model, our findings further demonstrated that a higher level of psychological resilience could protect individuals who suffered from the decline in economic status from developing more severe depressive symptoms. Third, the present study further supported the Richardson model of psychological resilience. The Richardson model mentions that when under colossal stress levels, individuals’ mental and physical equilibrium will be broken due to the suddenly increased risk factors (8). However, individuals with higher levels of protective factors to defend against negative influences will be able to maintain their equilibrium and protect their mental wellness. Psychological resilience, as an individual’s own quality and ability, represents an immaterial and internal competence that can be utilized to cope with crisis. Therefore, the power of psychological resilience in achieving the state of equilibrium was emphasized in this study. The lower level of depression demonstrated in individuals with higher psychological resilience as a personal protective resource championed the Richardson model regarding psychological equilibrium.

## Data availability statement

The original contributions presented in the study are included in the article/supplementary materials, further inquiries can be directed to the corresponding author.

## Author contributions

JW contributed to conception and design of the study. JH organized the database. LL performed the statistical analysis. YC, LL, and JH wrote sections of the manuscript. All authors contributed to the article and approved the submitted version.

## Conflict of interest

The authors declare that the research was conducted in the absence of any commercial or financial relationships that could be construed as a potential conflict of interest.

## Publisher’s note

All claims expressed in this article are solely those of the authors and do not necessarily represent those of their affiliated organizations, or those of the publisher, the editors and the reviewers. Any product that may be evaluated in this article, or claim that may be made by its manufacturer, is not guaranteed or endorsed by the publisher.
